# Blindness enhances auditory obstacle circumvention: Assessing echolocation, sensory substitution, and visual-based navigation

**DOI:** 10.1371/journal.pone.0175750

**Published:** 2017-04-13

**Authors:** Andrew J. Kolarik, Amy C. Scarfe, Brian C. J. Moore, Shahina Pardhan

**Affiliations:** 1 Vision and Eye Research Unit (VERU), Postgraduate Medical Institute, Anglia Ruskin University, Cambridge, United Kingdom; 2 Department of Psychology, University of Cambridge, Cambridge, United Kingdom; 3 Centre for the Study of the Senses, Institute of Philosophy, University of London, London, United Kingdom; 4 Department of Clinical Engineering, Medical Imaging and Medical Physics Directorate, Sheffield Teaching Hospitals NHS Foundation Trust, Sheffield, United Kingdom; Emory University, UNITED STATES

## Abstract

Performance for an obstacle circumvention task was assessed under conditions of visual, auditory only (using echolocation) and tactile (using a sensory substitution device, SSD) guidance. A Vicon motion capture system was used to measure human movement kinematics objectively. Ten normally sighted participants, 8 blind non-echolocators, and 1 blind expert echolocator navigated around a 0.6 x 2 m obstacle that was varied in position across trials, at the midline of the participant or 25 cm to the right or left. Although visual guidance was the most effective, participants successfully circumvented the obstacle in the majority of trials under auditory or SSD guidance. Using audition, blind non-echolocators navigated more effectively than blindfolded sighted individuals with fewer collisions, lower movement times, fewer velocity corrections and greater obstacle detection ranges. The blind expert echolocator displayed performance similar to or better than that for the other groups using audition, but was comparable to that for the other groups using the SSD. The generally better performance of blind than of sighted participants is consistent with the perceptual enhancement hypothesis that individuals with severe visual deficits develop improved auditory abilities to compensate for visual loss, here shown by faster, more fluid, and more accurate navigation around obstacles using sound.

## Introduction

For those who have lost their sight, information regarding the position of silent obstacles might be obtained using echoes from self-generated sounds, or using electronic sensory substitution device (SSD) travel aids, which provide information about the surrounding space by means of an intact modality, such as audition or touch [1]. Blind people are generally more sensitive to echoes than sighted people [2, 3], and often have improved echolocation abilities [4–9]. In the current study, we used an obstacle circumvention task to assess and compare navigation by sighted participants under three experimental conditions: auditory guidance using echolocation; a tactile SSD; and full vision. We also tested obstacle circumvention with early-onset blind participants, using auditory or SSD guidance.

According to the perceptual deficiency hypothesis, without vision to aid in calibrating audition, auditory spatial abilities may be worse for blind than for sighted individuals [10, 11]. Alternatively, blind individuals may have enhanced auditory spatial abilities as a result of extensive experience and reliance on auditory information [12], and because compensatory processes, such as cortical reorganization, may have enhanced auditory spatial performance in certain conditions [13]. Both views have received support in previous studies [3, 14–17], for a review, see [18] and one of the aims of the current work was to test whether blindness results in perceptual deficiency or enhancement when navigating around an obstacle using sound. Some blind people are able to use echolocation, by generating click-like sounds and listening to the returning echoes, which provide information about the spatial layout of the environment [19, 20]. Sighted people can also be trained to echolocate [21–23], and self-generated sound can guide locomotion when vision is absent (see [24] for a review). Wallmeier and Wiegrebe [25] reported that sighted and blind individuals could echolocate to accurately adjust the orientation of the body, and move towards a chosen direction. Furthermore, expertise gained by blind echolocators can lead to excellent spatial abilities in order to guide locomotion. Thaler, et al. [26] tested two blind expert echolocators who used clicks to explore cities, and also for hiking, mountain biking and playing basketball. McCarty and Worchel [27] described a blind echolocator who used clicks to avoid obstacles when riding a bicycle. Fiehler, et al. [28] found that blind expert echolocators were better able than blind and sighted non-echolocators to use echolocation clicks, previously recorded when walking along a corridor, to identify whether the corridor’s direction was straight ahead, leftwards or rightwards. Teng, et al. [19] found that blind expert echolocators achieved a relatively high degree of precision when determining the location of objects, which was similar to that found in the visual periphery of sighted individuals.

Overall, these results suggest superior echolocation acuity for blind echolocators, which may lead to superior navigation using sound. However, echolocation abilities within the blind population demonstrate substantial individual variability. While some studies show that good echoic auditory abilities are associated with blindness even without extensive training [2, 3, 6], other studies have suggested that it is echolocation expertise rather than blindness that drives performance, as blind non-echolocators performed similarly to sighted participants and performed more poorly than blind expert echolocators in various tasks including using sound echoes to determine 2-D shape [29], using echolocation to correctly identify physical size independently of distance (size constancy) [30], and in auditory spatial bisection tasks [31]. In the current study, obstacle circumvention performance for a group of blind non-echolocators was compared to performance for a normally sighted group, and a single blind expert echolocator.

Previous work utilizing a classical obstacle approach and detection task has shown that sound can guide locomotion in both blind people [32–34] as well as blindfolded sighted people [32, 35–37]. In these studies, participants reported when they first detected a large obstacle (such as a wall) in their path. They then approached as close as they could while avoiding collision, and stopped in front of the obstacle. Relatively few collisions were made, and blind participants generally performed better than blindfolded sighted participants. In general, blind participants detected obstacles at a similar or increased range compared to sighted controls and made fewer collisions [32]. Kolarik, et al. [24] assessed obstacle circumvention performance in blindfolded sighted people using self-generated clicks, and showed that participants could successfully navigate around the obstacle on 67% of the trials. However, movement times, velocity corrections, and buffer space (clearance between the shoulder and the obstacle) were greater than when participants navigated using vision. In addition, under visual guidance participants passed the obstacle on the side affording most space, but they were not able to do this using echolocation. However, performance for blind participants performing a similar obstacle circumvention task has not yet been investigated. This was the main aim of the current study.

Another form of locomotor guidance for use by participants without vision is offered by SSDs. Some, referred to in the current paper as “echoic SSDs,” use ultrasound echoes to detect objects and then provide a tactile or auditory cue regarding the distance of the object. Other “visual pattern SSDs” transform visual signals into sound or tactile information [38]. For early-onset blind participants, an echoic SSD improved judgements of obstacle direction and distance [39]. Blind and blindfolded sighted participants have also been shown to use a visual pattern SSD named the tongue display unit (TDU) to negotiate an obstacle course [40], and Kupers, et al. [41] showed that blind participants were more accurate than sighted participants in using the TDU in a virtual route recognition task. Hughes [42] showed that blindfolded normally sighted participants were able to use an echoic SSD to judge the passability of narrow apertures, and Kolarik, et al. [43] found that blindfolded sighted participants navigating under echoic SSD guidance made appropriate shoulder rotations to allow them to pass through narrow apertures safely. Kolarik, et al. [44] also investigated obstacle circumvention by blindfolded sighted participants using an echoic SSD, and showed that movement times, velocity corrections, and buffer space were greater than when participants navigated using full vision, and that participants generally passed the obstacle on the side affording most space. However, the effects of blindness on obstacle circumvention kinematics under SSD guidance have not previously been investigated, and were examined in the current study.

Rosenblum, et al. [45] pointed out that relatively few studies have directly compared behaviorally relevant performance across modalities, to determine if different senses can guide behavior in a comparable manner. Obstacle circumvention may be particularly behaviorally relevant, as detecting and walking around an obstacle in the path of travel is one of the most commonly encountered tasks of everyday locomotion [46, 47]. Furthermore, the task extends the classic obstacle approach and detection task described above, requiring sound or tactile input to be used not only to estimate the distance of an obstacle, but also to accurately perceive the location of the edges and navigate around the obstacle without collision. The current study is the first that we are aware of to formally assess kinematic indices for blind non-echolocators and a blind expert echolocator using echolocation or an SSD during obstacle circumvention. The kinematic indices measured included buffer space, movement times, and number of velocity corrections. Increased buffer space is indicative of increased caution by allowing a greater margin between the body and obstacle, at the cost of expending greater energy. Movement time provides practical information regarding how long is needed to determine the size and location of an obstacle. The number of velocity corrections provides information about how fluid the movement is under different forms of sensory guidance, as frequently stopping and starting would increase energy consumption and reduce efficiency. These measures provide an indication of how practical sound echoes are compared to vision for navigation outside of the laboratory. As for previous studies [24, 44], locomotion with full vision in normally sighted participants provided a baseline for performance.

The current study extends previous work by Kolarik, et al., that investigated the kinematics of obstacle circumvention for blindfolded normally-sighted participants using echolocation [24] and SSD guidance [44], by using similar methods to investigate obstacle circumvention by blind non-echolocators and an expert blind echolocator. The findings are intended to increase our understanding of how blind individuals navigate in real-world environments, and to objectively measure obstacle circumvention performance using auditory, tactile and visual guidance in sighted blindfolded participants, and using auditory and tactile guidance in blind non-echolocators, and a blind expert echolocator. In addition, a “no-click” auditory guidance control experiment was used to assess obstacle circumvention based on footfalls on the carpet or ambient sound, rather than self-generated clicks or SSD information, for sighted blindfolded participants, blind non-echolocators, and a blind expert echolocator [24].

The following hypotheses were tested: 1) If enhanced abilities to use sound echoes following blindness [2, 3] can be used to improve navigation, and blindness is the primary factor that drives the ability to use auditory cues for navigation, then blind non-echolocators should show better locomotion performance than the sighted group under auditory guidance. This should result in fewer velocity corrections, shorter movement times, smaller buffer space and fewer impacts, consistent with the perceptual enhancement hypothesis; 2) On the other hand, if echolocation expertise [29–31] is the primary factor driving the ability to use auditory cues for navigation, then only the blind expert echolocator should show better locomotion performance under auditory guidance, and the blind non-echolocators should perform similarly to or even more poorly than the other groups, consistent with the perceptual deficiency hypothesis; 3) Navigation performance should be better under visual guidance than under auditory or SSD guidance.

## Methods

The apparatus, data acquisition, and procedure (described below) were the same as used by Kolarik, et al. [24] for evaluating the kinematics of obstacle circumvention for sighted participants using auditory guidance and full vision, and for sighted participants using SSD guidance and full vision [44]. These methods are applicable to both normally sighted and visually impaired participants.

### Participants

There were 19 participants, comprising a normally sighted group (n = 10, 5 females, mean age 37 yrs, range 22–59 yrs), a blind non-echolocator group (n = 8, 5 females, mean age 41 yrs, range 25–54 yrs, B1-8 in Table 1), and a blind expert echolocator (female, aged 20 yrs, BE in Table 1). Data for a number of sighted participants tested in previous work, in our laboratory under identical conditions, under auditory guidance (n = 7, [24]) or SSD guidance (n = 9, [44]) were included in the data for the current normally sighted group, and additional sighted participants were included in order to age-match this group with the blind non-echolocating group. For the “no-click” control experiment, all blind participants and 8 sighted participants took part (3 females, mean age 37 yrs, range 29–52 yrs, 4 of whom took part in the main experiment). All blind participants were either totally blind or had some light perception only, and fell into categories 4–5 of the World Health Organization classification [48]. All were early-onset blind, defined here as having lost their sight between birth and 5 yrs of age (Table 1). The sighted participants all reported normal or corrected-to-normal vision. All participants had normal hearing. Their pure tone thresholds were less than or equal to 25 dB HL for all audiometric frequencies up to 8 kHz, measured with an Interacoustics AS608 audiometer using methods described by the British Society of Audiology [49]. None of the normally sighted or blind non-echolocators had received prior echolocation training to perceive objects or to navigate in their daily lives. The BE echolocator used echolocation in daily life, and had received training at a formal course teaching echolocation skills. None of the participants had any previous experience using tactile echoic SSDs. All participants were right handed except for BE and one sighted participant. All participants gave written informed consent following an explanation of the nature and any possible consequences of taking part in the experiments. The study was approved by the Anglia Ruskin University Ethics committee, and the experiments followed the tenets of the Declaration of Helsinki.

**Table 1:** Details of blind participants. B1-8 were non-echolocators. BE was an expert echolocator.

		Age, age of onset of vision loss (yrs)	Gender	Cause of vision loss	Visual status, WHO category
B1	54, 5		F	Macular degeneration	Light perception, 4
B2	48, 5		M	Stickler's Syndrome, retinal detachment	No light perception, 5
B3	54, 1.5		F	Glaucoma	No light perception, 5
B4	41, 1		F	Retinoblastoma	No light perception, 5
B5	26, 2		F	Norrie disease	Light perception, 4
B6	25, 3		M	Retinoblastoma	No light perception, 5
B7	42, birth		F	Retinopathy of prematurity	No light perception, 5
B8	39, 1		M	Retinoblastoma	Light perception, 4
BE	20, 2.5		F	Retinoblastoma	No light perception, 5

### Apparatus and data acquisition

**Fig 1:**
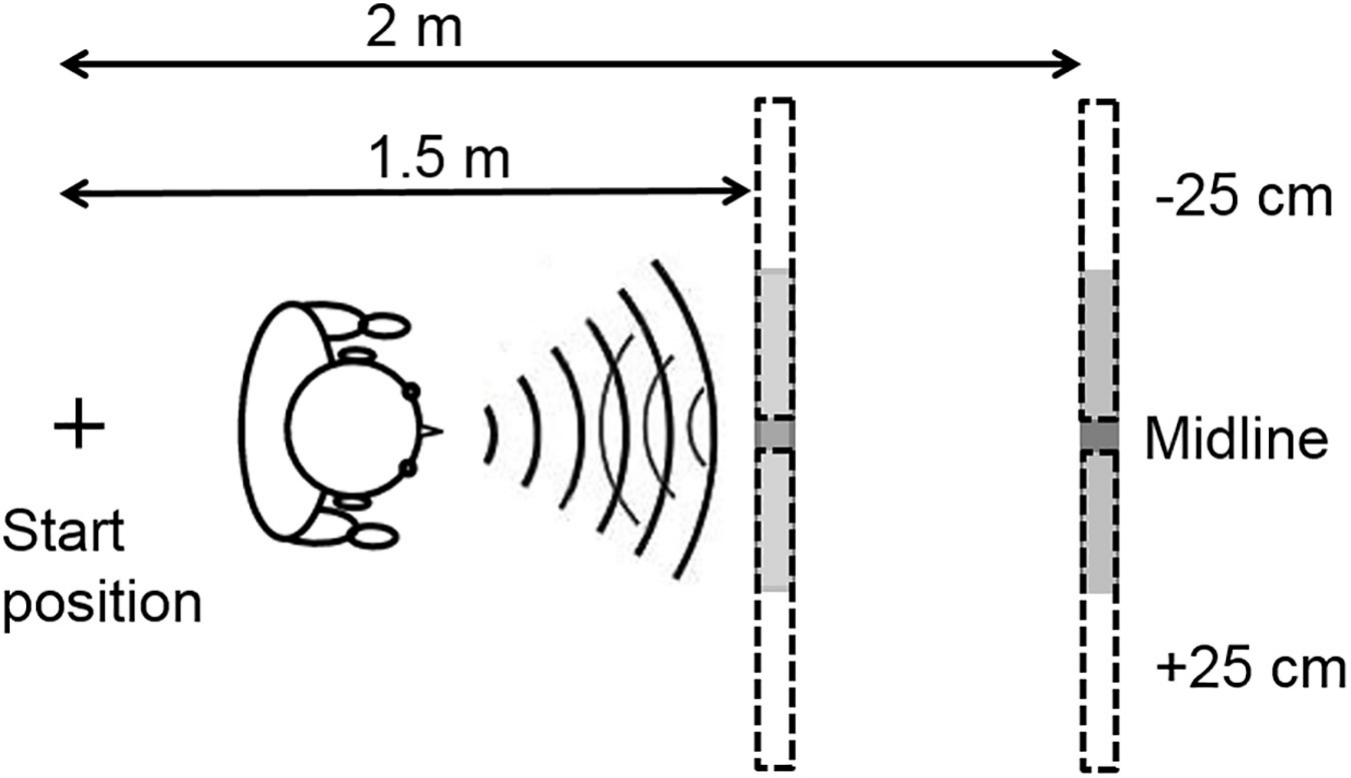
Experimental layout. The obstacle was 0.6-m wide, located directly ahead of the participant on the midline (shaded bar), or ±25 cm leftwards or rightwards (bars marked by dashed lines). Two approach distances were used (1.5 and 2 m).

The experiments took place in a quiet room (ambient sound level of approximately 36 dBA) measuring 5.7 × 3.5 m, height 2.8 m. The room had a tiled ceiling and painted walls, and a carpet covered the floor. The obstacles used for testing were rectangular, movable, flat, and made of wood. Smooth reflective aluminum foil was used to cover the obstacles to achieve high reflectivity, as used by Arnott, et al. [50]. However, the reflective foil also produced near-specular sound reflections, which may have made the obstacle harder to accurately locate than would be the case for a wood surface. For static training, a practice small obstacle, width 0.5 m × height 0.34 m, with a thickness of 2 cm was utilized. For dynamic training and testing, a large obstacle mounted on castors was utilized, width 0.6 m × height 2 m, with a thickness of 0.6 cm, with a small plastic frame at the bottom on the side of the obstacle not facing the participant. The obstacle was placed near the middle of the room, to prevent SSD reflections from surfaces apart from the obstacle used in the experiment. Fig 1 shows the layout of the experimental set up. The SSD, the Miniguide [51], provided spatial information via a tactile signal, so that if an obstacle was present the aid transmitted vibrations to the hand that held the device. The SSD vibrated at a rate that was proportional to the distance between the obstacle and the SSD. The operable range of the device was set to 1 m, and participants held the device in their dominant hand perpendicular to the body with the device pointed straight ahead, while tucking their elbow against their side, as reported in Kolarik, et al. [43].

Trials in which the SSD or a part of the body of the participant collided with the obstacle were noted by the experimenter, and these were not used in the final analyses of the kinematic indices. The side (on the right or left of the obstacle) used by the participant to avoid the obstacle was also noted by the experimenter. As described in detail previously [24, 44], an 8-camera motion capture system (Vicon Bonita; Oxford Metrics) was used to collect three-dimensional kinematic data at a rate of 50-Hz. Retro-reflective, spherical markers were placed bilaterally at the following locations on the participant’s body: the posterior aspect of the calcanei, the acromio-clavicular joint, the most distal, superior aspect of the 1st toe, the most distal, superior aspect of the 5th toe, and antero-lateral and postero-lateral aspects of the head. Single markers were attached on the sternum and the posterior aspect of the dominant hand. Three markers were attached to the front of the obstacle, to define the obstacle location and width in the coordinate system of the laboratory. Kinematic data were processed using Vicon Nexus (Oxford Metrics) and the cross-validatory quintic spline smoothing routine was used to filter the marker trajectory data. ‘Smoothing’ options were set at a predicted mean squared error value of 10. Custom written Visual Basic scripts were used to evaluate key variables involving obstacle circumvention, or the experimenter recorded these (see Table 2). When the participant accelerated or decelerated in the anterior/posterior direction, a velocity correction was recorded. This was required to last for 50 frames (1 second) to avoid including very small velocity fluctuations. Slowing down and speeding up, and stopping and starting were all recorded as velocity corrections. To be counted as a velocity correction, a change in trajectory was required to involve a forward acceleration or deceleration. A side step without slowing down in the anterior/posterior direction was not counted. If participants stopped, side stepped and then moved forward, this was recorded as two velocity corrections (the first for decelerating prior to side stepping, and the second for accelerating after side stepping).

**Table 2:** Dependent variables. These were also described in detail in previous studies of obstacle circumvention [24, 44].

Variable	
Obstacle-present trials	
Buffer space	The medio-lateral distance between the obstacle marker and the shoulder marker at the point of passing the obstacle marker (determined as the point where the shoulder marker passed the marker attached to the front aspect of the obstacle).
Movement time	Time to complete the movement, measured from when the obstacle marker was 1 m distant from the sternum marker in the anterior-posterior direction, until the obstacle marker was passed.
Velocity corrections	Number of changes in forward velocity (changes from acceleration to deceleration or vice versa), measured from when the obstacle marker was 1 m from the sternum marker until the point of passing the obstacle marker. For additional information, see main text.
Obstacle detections	Auditory guidance trials when the participant raised their hand marker to indicate obstacle perception, recorded by the experimenter.
Obstacle detection range	The anterior-posterior distance between the obstacle marker and the participant’s sternum marker. This was measured at the time when the participant raised their hand marker, indicating perception of the obstacle.
Collisions	Trials where a collision occurred between any part of the participant’s body or the SSD and the obstacle, recorded by the experimenter.
Side of obstacle avoidance	The experimenter recorded the side of avoidance of the obstacle (passing the obstacle on the left or right).
False perceptions on obstacle-absent trials	The number of obstacle-absent auditory guidance trials in which the participant raised their hand marker, incorrectly indicating perception of the obstacle.

### Procedures

All participants took part in the auditory guidance and SSD conditions (order randomized and counterbalanced across participants). For normally sighted participants, the visual guidance condition was performed last [24, 43], so that participants did not become familiar with the different obstacle placements. Except in the visual guidance condition, all participants wore close-fitting blindfolds, for consistency across participants and to prevent use of residual light perception by some of the blind non-echolocator group (BE had no residual light perception).

Participants received static and dynamic training at the start of the auditory guidance condition. This has previously been shown to allow sighted participants to become sufficiently familiar with echolocation to navigate around an obstacle without collision in the majority of trials [24]. It allowed participants to practice producing mouth clicks and to listen to them during locomotion. For the static training, participants sat in the middle of the room with the small obstacle positioned on a table directly in front of them 25 cm away and at head height. Participants made mouth clicks to detect the presence of the obstacle, which was randomly present or absent. Feedback was provided. The duration of the static auditory training was at least 15 minutes. For the first 5 minutes the sighted participants were instructed to keep their eyes open, for the next 5 minutes they were instructed to attempt the task with eyes closed, and for the last 5 minutes they wore a blindfold. Blind participants were blindfolded for the last five minutes. For sighted participants, visual feedback was allowed in the first two stages, following previous work [24] and pilot testing indicating that this group often showed initial difficulties associating the echoes with the presence or absence of the obstacle, whereas blind participants did not report any difficulties. All participants reported that they felt that they were able to hear the changes in echoes sufficiently following static training to move onto the next phase of training.

In the dynamic auditory training phase, participants practiced moving around the large obstacle, using mouth clicks to perceive its location. The approach distance was 1.75 m, and training lasted for at least 15 minutes. As for static training, for the first 5 minutes the sighted participants were allowed to keep their eyes open, followed by five minutes in which they were encouraged to close their eyes, and for the last five minutes they were blindfolded. Blind participants wore a blindfold for the last five minutes. The obstacle center was placed on the midline directly straight ahead of the participant for the first 10 minutes. During the last 5 minutes of training, when the participants were blindfolded, the obstacle’s location was varied randomly relative to the participant: at the midline or 25 cm to the right or left. After training, all participants reported that they were sufficiently familiar with hearing the changes in echoes to circumvent an obstacle. To test whether participants were able to detect obstacles using echolocation prior to the auditory testing phase, blindfolded participants stood 25 cm from and facing the position where the large obstacle might be placed, made mouth clicks, and were asked to report whether they perceived the obstacle to be present or absent for twenty trials. For half of the trials the obstacle was present, and for the other half it was removed (randomized). Mean detection performance was 84% (SD = 12%) for the sighted group, 97% (SD = 6%) for the blind non-echolocators, and 100% for BE. All participants scored 70% or higher.

In the auditory testing phase, participants were instructed that they would wear a blindfold, should walk straight forward while making mouth clicks, and should report if they perceived the large obstacle to be present. They were instructed to move in a straight line until first perceiving the obstacle, to indicate this by raising their dominant hand, as used for the obstacle approach and detection task in previous work [32, 37], and then to circumnavigate the obstacle without touching it. Participants were instructed not to use their hands to touch the obstacle, and they were told that the obstacle would be absent in some trials. When the participant had moved past the obstacle or if the participant touched or collided with the obstacle, the trial was terminated. The obstacle was positioned randomly either on the midline, or 25 cm rightwards or leftwards. The approach distance was either 1.5 or 2 m (randomized). The approach distances were less than previously tested for visual obstacle circumvention e.g. 5 m [46, 47], but not for auditory [24] or SSD-guided [44] obstacle circumvention tasks. Pilot data indicated that participants generally stood stationary a short distance before the obstacle, and scanned it using audition (or the SSD in the SSD guidance condition), as was observed in a previous study for participants moving through apertures using an SSD [43].

Three trials were performed for each obstacle position. In total, 24 trials were completed, including six no-obstacle ‘catch’ trials [33, 34, 52]. Ear-defender headphones were used to occlude participants’ ears between each trial. The experimenter signaled the start of the trial by tapping on the participant’s shoulder.

In the SSD condition, participants performed an SSD dynamic training phase followed by an SSD testing phase. The SSD training phase was similar to the auditory guidance training, except that practice with the small obstacle and static detection of the large obstacle were not included, as in this condition participants did not need to become familiar with producing sounds and the device always allowed them to detect the obstacle if it was present. In previous experiments that assessed the use of an echoic SSD for aperture navigation, training with the device lasted approximately 5 minutes [42, 53]. To provide greater training with the SSD than provided in these experiments, participants practiced circumventing the large obstacle using the SSD from an approach distance of 1.75 m, for at least 15 minutes. Following dynamic SSD training, participants performed the SSD testing phase. This was similar to the auditory guidance testing phase, except that participants used the SSD for obstacle circumvention instead of producing mouth clicks and wore ear-defender headphones during the trials, as reported by Kolarik et al. [44], to avoid sound from footfalls on the carpet or ambient auditory information being audible.

Normally sighted participants performed a visual guidance condition similar to that tested by Hackney, et al. [46]. The visual guidance condition was the same as the testing phase of the other conditions, except that the SSD was not used and mouth clicks were not produced. Ear-defender headphones were worn, and participants were blindfolded between trials only.

At the start of each trial for the auditory and SSD dynamic training and testing phases, a removable plastic box was used so that participants could align their feet facing forward along the edge of the box. The experimenter always took participants back to the starting point at the end of each trial, and they placed themselves in the same place to the right of the starting point against the wall throughout the trial. Except when required to make mouth clicks, the experimenter and participant were silent throughout testing. The participants did not receive feedback during the testing phases. Data were obtained in a single session lasting approximately 2.5 hours (including breaks) for the blind participants, and 3 hours for the sighted participants (the duration was longer as they also performed the visual guidance condition).

For the “no-click” control experiment, participants did not produce mouth clicks, and completed seven trials (one for each obstacle position and one with the obstacle absent). Otherwise the procedure was identical to that for the testing phase of the auditory-guidance condition of the main experiment. Data were collected in a single session lasting approximately 20 minutes.

### Statistical analyses

To compare the performance of the sighted and the blind non-echolocator groups, unless otherwise specified, repeated-measures analyses of variance (ANOVAs) were utilized to analyze how movement time, buffer space, side of obstacle avoidance (right or left), and number of velocity corrections were influenced by visual status (sighted or blind non-echolocators, a between-subjects factor), guidance condition (audition, SSD and vision), and repetition (one, two and three) (within-subjects factors). The significance level was *p*<0.05. Following preliminary analyses showing that scores for all measures did not vary significantly (*p*>0.05) with obstacle lateral location or distance, the results for the three locations (with the exception of the side of obstacle avoidance analysis) and two distances were pooled. Prior to analysis, proportional data for side of avoidance were subjected to arcsine transformation [54]. Bonferroni correction was used for post hoc analyses.

Data from trials where collisions with the obstacle occurred were not used in the final analyses of the kinematic indices. It is possible that a difference across groups in the numbers of trials with collisions might have affected the results for the other parameters. However we believe that any such effect is likely to be small, since there were 18 trials for each condition where collisions might have occurred and the obstacle was passed without collision in the majority of those trials for each group. Thus, it is likely that sufficient trials were included in the analyses to avoid potential biases.

The performance of BE was compared to that of the sighted and the blind non-echolocator groups using *t*-tests as modified by Crawford et al. [55, 56], and as described by Vercillo, et al. [31]. The modified *t*-tests tested the hypothesis that scores for BE did not originate from scores from a comparison population. The null hypothesis was that the score for a given measure for BE came from a distribution that had the same mean and variance as for the comparison group [55, 56]. In this way, scores for the comparison sample were treated as statistics instead of population parameters. If the value of *t* obtained from the test was below the negative one-tailed 5% critical value, then the null hypothesis was rejected and it was concluded that BE’s score was not an observation from the population of scores for the comparison group. One-tailed tests were used as it was hypothesized that BE would perform better than the other participant groups.

## Results

### Obstacle detections and collisions

For participant data, see S1 File. Under auditory guidance, five trials for the sighted group and three trials for the blind non-echolocators were discarded from the analyses, as the participants did not start the trial facing directly to the front.

Table 3 summarizes the percentage of trials on which the obstacle was detected, trials where no collision occurred, and false reports, for auditory guidance, SSD guidance, and the “no-click” control experiment. In the main experiment, all groups detected the obstacle in the majority of trials. Under auditory guidance when the obstacle was present the obstacle was detected on 75% of trials for the normally sighted group, 96% of trials for the blind non-echolocators, and 100% of trials for BE. Participants always detected the obstacle under SSD guidance. Under auditory guidance when the obstacle was present there were no collisions on 58% of trials for the normally sighted group, 88% of trials for the blind non-echolocators, and 94% of trials for BE. Under SSD guidance when the obstacle was present there were no collisions on 93% of trials for the normally sighted participants, 90% of trials for the blind non-echolocators, and 83% of trials for BE. As expected, there were no collisions when navigating using full vision. In the auditory guidance condition, the percentage of false reports of the obstacle being present were 17% for the normally sighted group, 27% for the blind non-echolocators, and 0% for BE. Sighted participants collided with the obstacle significantly more than the blind non-echolocators under auditory guidance [χ^2^ (1) = 34.02, *p* = 0.001], but not under SSD guidance [χ^2^ (1) = 1.47, ns].

**Table 3:** Summary of obstacle detection, collision and false reports. The percentage of obstacle-present trials is reported in which the obstacle was detected, or for which collisions did not occur. The percentage of false reports of the obstacle being present for obstacle-absent trials is also reported. Performance under auditory guidance, SSD guidance, and for the “no-click” control experiment is reported. Under visual guidance, the obstacle was always detected, no collisions occurred, and there were no false reports.

	Normally sighted (mean, SD)	Blind non-echolocators (mean, SD)	BE (mean)
**% of trials in which the obstacle was detected**			
Auditory guidance	75 (18)	96 (4)	100
SSD	100 (0)	100 (0)	100
“no-click” control experiment	21 (31)	81 (21)	83
**% of trials where collision did not occur**			
Auditory guidance	58 (21)	88 (7)	94
SSD	93 (8)	90 (15)	83
“no-click” control experiment	11 (23)	69 (19)	50
**% of false reports of obstacle being present**			
Auditory guidance	17 (22)	27 (23)	0
SSD	0 (0)	0 (0)	0
“no-click” control experiment	0 (0)	25 (46)	0

For the “no-click” control experiment, the percentages of trials on which the obstacle was detected were: sighted group, 21%; blind non-echolocators, 81%; BE, 83%. The percentages of trials on which the obstacle was circumvented without collision were: sighted group, 11%; blind non-echolocators, 69%; BE, 50%. False perceptions for obstacle-absent trials were: sighted, 0%; blind non-echolocators: 25%; BE: 0%. These results show that although the blind participants were often able to detect and circumvent the obstacle without making mouth clicks, sighted participants often failed to detect the obstacle and almost always collided with it, and performance for all groups was lower than performance with mouth clicks. The results are consistent with the finding of Supa, et al. [32] that both blind and sighted participants showed lower performance on an obstacle approach and detection task when using sound from stockinged feet on a carpet runner than when using sound from shoes on a hardwood floor.

**Fig 2:**
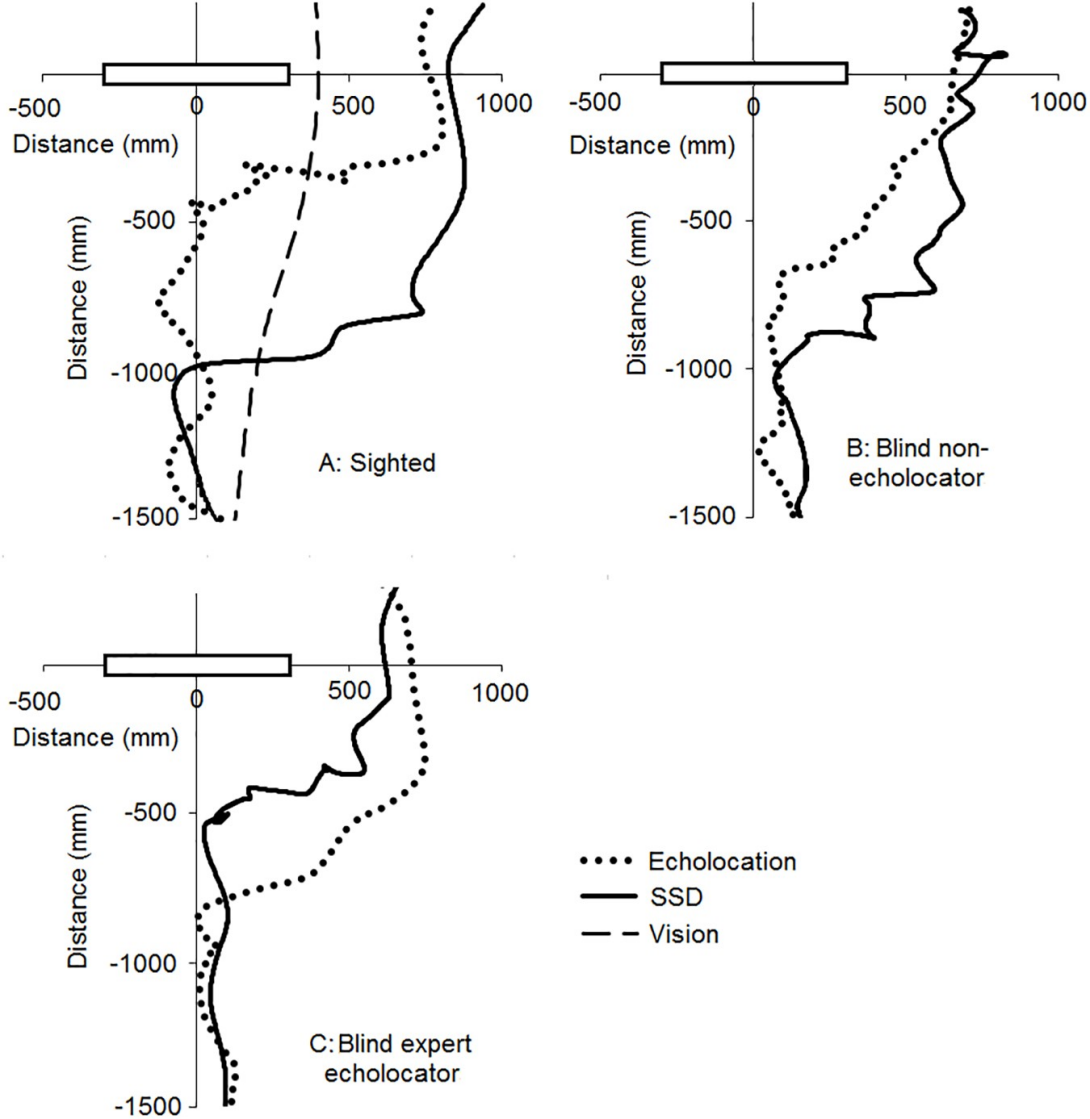
**Representative participant trajectories under auditory guidance using echolocation (dotted line), SSD guidance (solid line) and visual guidance (dashed line) for a sighted participant (top left panel A), a blind non-echolocator (top right panel B), and the blind expert echolocator (BE, bottom left panel C).** The obstacle is shown by the black rectangle. Data are shown for the left shoulder marker, for an approach distance of 1.5 m. The obstacle was placed 25 cm to the participant’s left.

Fig 2 shows trajectories for a representative participant from each group under auditory guidance (dotted line), SSD guidance (solid line) and visual guidance (dashed line). The obstacle’s lateral position was 25 cm to the left of the participant. For sighted participants (panel A) and blind non-echolocators (panel B), participants generally moved closer to the obstacle under auditory guidance than under SSD guidance before showing distinct deviations from moving straight ahead. However, the expert blind echolocator (panel C) generally moved closer to obstacle under SSD guidance than under auditory guidance before substantially deviating from the straight-ahead direction. This is consistent with the difference in operable range for detecting the obstacle across different guidance conditions, as participants only substantially deviated from the midline when the obstacle was within detectable range. The operating range of the SSD was set to 1 m, and participants under SSD guidance deviated substantially from straight ahead when the obstacle was within the operative range. Under auditory guidance, the mean echolocation detection range was less than 1 m for the sighted and blind non-echolocators, whereas the mean detection range of BE was greater than 1 m (see Echolocation detection range section below). Under visual guidance, participants deviated from straight ahead immediately when movement was initiated. In no-obstacle catch trials, participants moved approximately straight ahead in all conditions, showing that substantial deviation from the midline only occurred in the presence of an obstacle.

### Movement times

**Fig 3:**
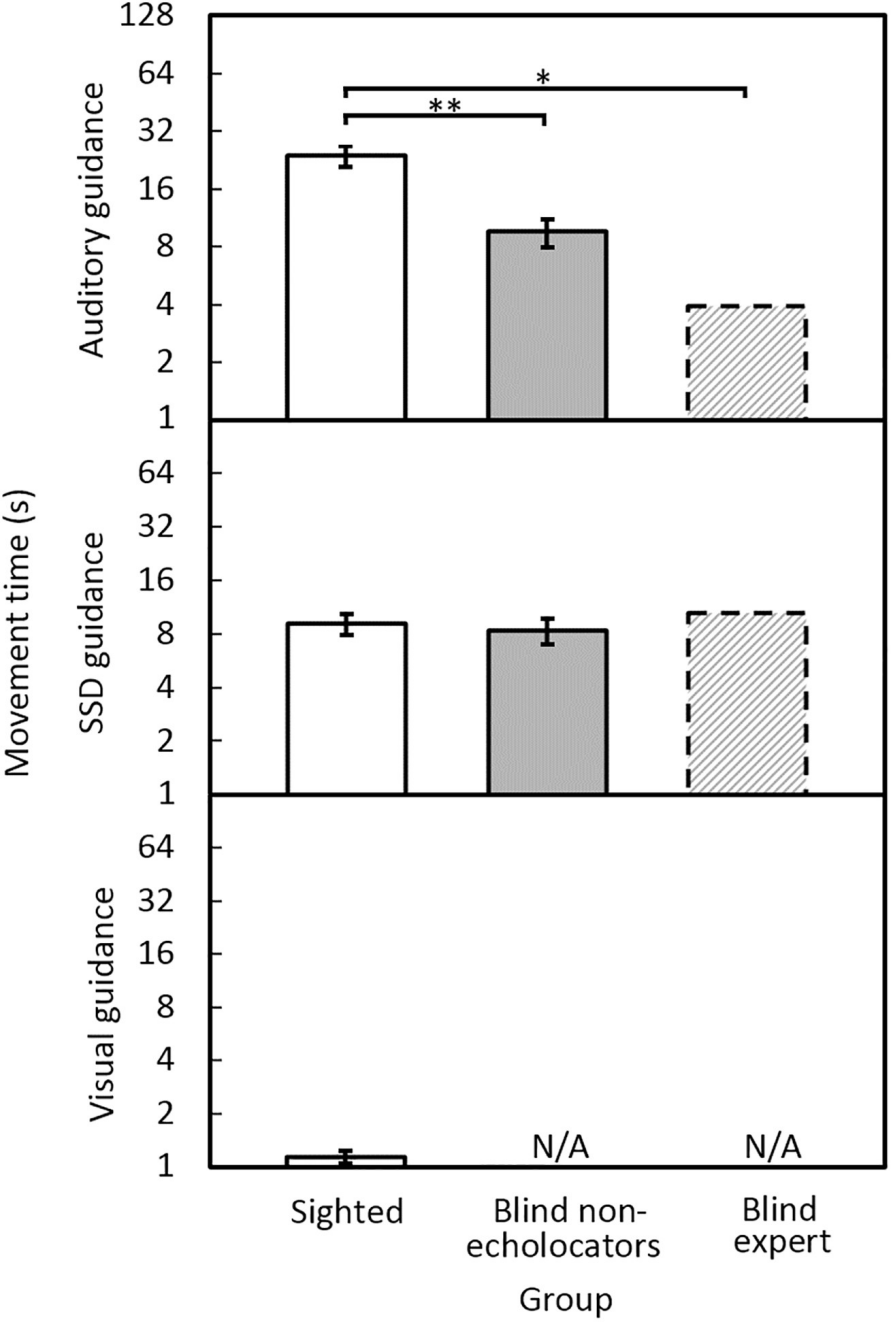
Mean movement time to circumvent the obstacle under auditory guidance (upper panel), SSD guidance (middle panel), or visual guidance (lower panel), for sighted participants (open bars), blind non-echolocators (grey bars), and BE (dashed bars). In this and subsequent figures, 0 indicates where there was no effect (e.g. zero collisions), N/A marks when the group did not participate, error bars represent ±1 standard error, and asterisks indicate significant differences, * <0.05, ** <0.01. The y axis is logarithmic.

Fig 3 shows the mean movement times required to circumvent the obstacle. Under auditory guidance, the times were shortest for BE, larger for the blind non-echolocating group, and largest for the sighted group. Under SSD guidance, the times were similar for all groups. As anticipated, movement times for the sighted group were shortest under visual guidance. For sighted participants, the mean movement times were 23 s under auditory guidance, 9 s using the SSD, and 1.1 s using vision. For blind non-echolocators the corresponding times were 10 s under auditory guidance and 8 s under SSD guidance. For BE, the times were 4 s using auditory guidance, and 11 s under SSD guidance. The blind non-echolocators were significantly faster than the sighted group under auditory guidance [*F*(1, 16) = 18.22, *p* = 0.001], but not SSD guidance [*F*(1, 16) = 0.20, ns]. There was no effect of repetition. Movement times for the sighted group under visual guidance were significantly faster than for the blind non-echolocators under auditory guidance [*F*(1, 16) = 38.56, *p* = 0.001] or SSD guidance [*F*(1, 16) = 41.03, *p* = 0.001]. Modified *t*-tests showed that under auditory guidance BE circumvented the obstacle in a significantly shorter time than the sighted participants (*t* = 2.21, *p* = 0.03) but not the blind non-echolocators (*t* = 1.23, ns).

### Velocity corrections

**Fig 4:**
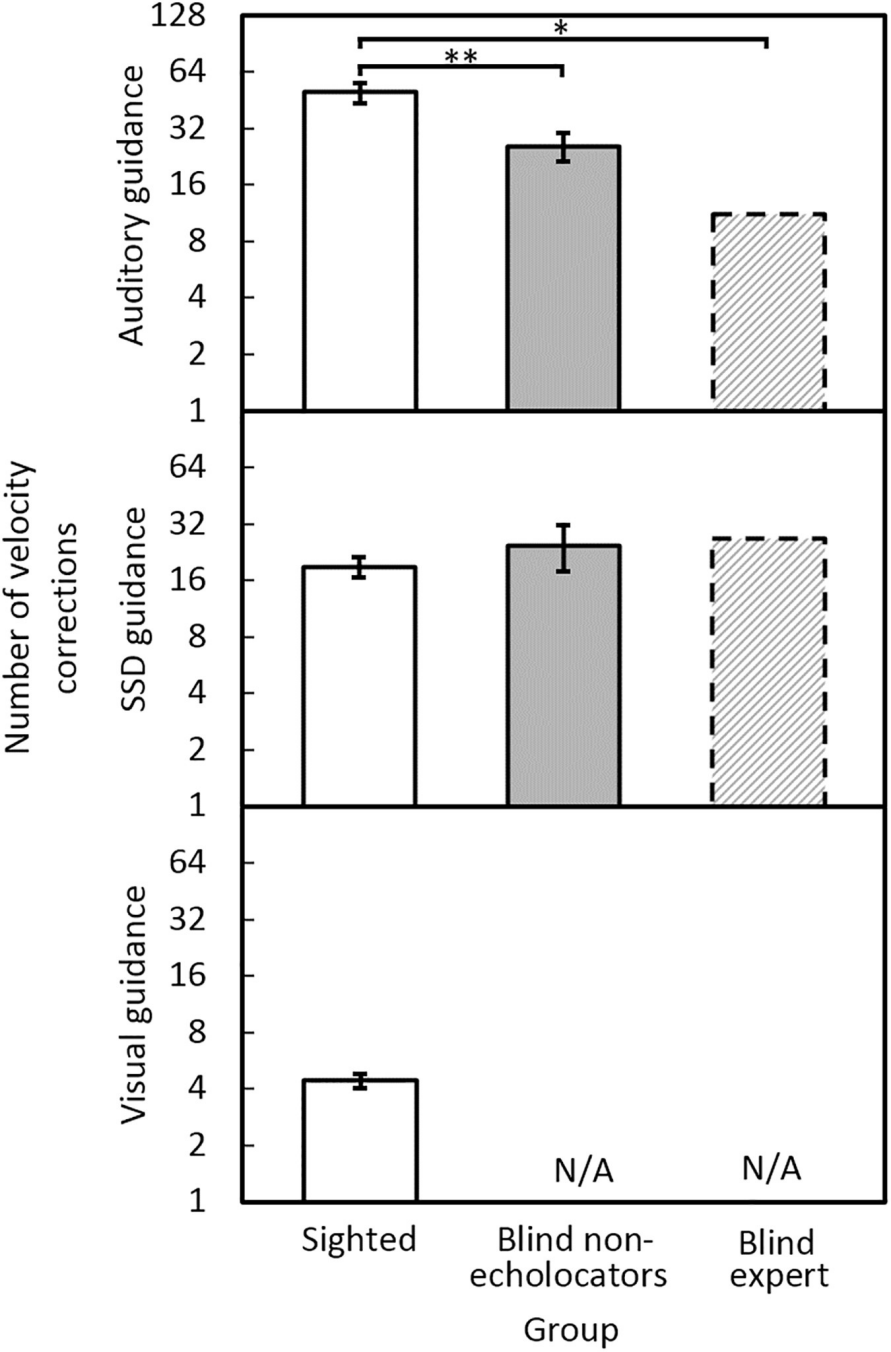
Mean number of velocity corrections under auditory guidance (upper panel), SSD guidance (middle panel), and vision (lower panel), for sighted participants (open bars), blind non-echolocators (grey bars), and the BE echolocator (dashed bars). The y axis is logarithmic.

Fig 4 shows the mean number of velocity corrections for each group and condition. Under auditory guidance, the BE echolocator made the fewest velocity corrections and the sighted group made the most. Under SSD guidance, all groups made similar numbers of velocity corrections. Fewest velocity corrections were made under visual guidance. For sighted participants, the mean velocity corrections were 50 using auditory guidance; 19 using the SSD; and 4 using vision. For blind non-echolocators, the mean velocity corrections were 26 using auditory guidance; and 25 under SSD guidance. For BE, the mean velocity corrections were 11 under auditory guidance and 27 under SSD guidance. The blind non-echolocators made significantly fewer velocity corrections than the sighted group under auditory guidance [*F*(1, 16) = 11.0, *p* = 0.004] but not SSD guidance [*F*(1, 16) = 0.93, ns]. There was no effect of repetition. Mean numbers of velocity corrections for the sighted group under visual guidance were significantly fewer than for the blind non-echolocators under auditory guidance [*F*(1, 16) = 34.7, *p* = 0.001] or SSD guidance [*F*(1, 16) = 13.7, *p* = 0.002]. Modified *t*-tests showed that, on average under auditory guidance, BE made significantly fewer velocity corrections than the sighted participants (*t* = 2.10, *p* = 0.03) but not the blind non-echolocators (*t* = 1.20, ns).

### Buffer space

**Fig 5:**
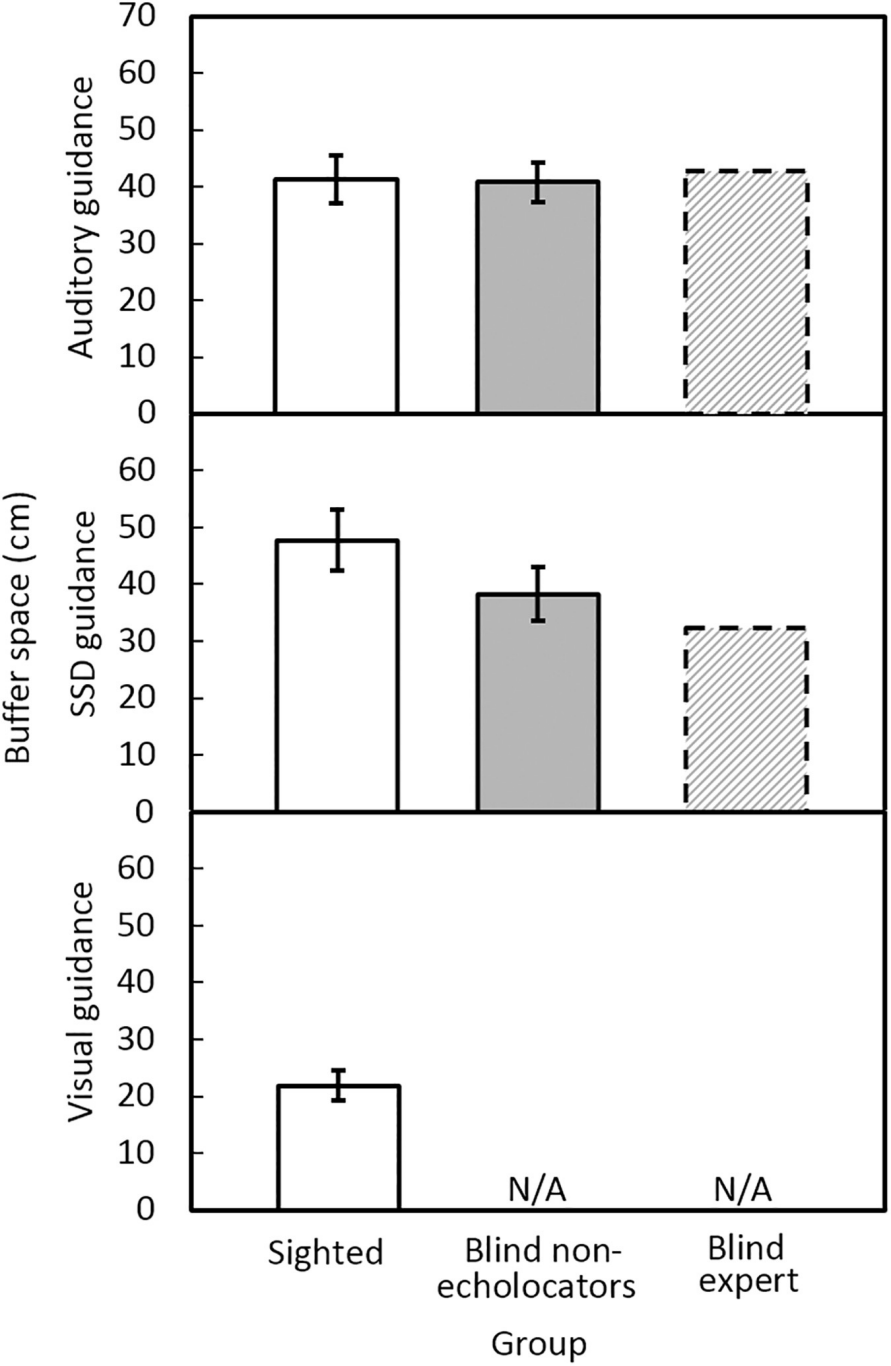
Mean buffer space at the time of crossing under auditory guidance (upper panel), SSD guidance (middle panel), and visual guidance (lower panel), for sighted participants (open bars), blind non-echolocators (grey bars), and the BE echolocator (dashed bars).

Fig 5 shows the mean buffer space for each condition and group. The buffer space was smallest under visual guidance. For sighted participants, mean buffer spaces were 41 cm using auditory guidance; 48 cm using the SSD; 22 cm for vision. For blind non-echolocators the mean buffer spaces were 41 cm using auditory guidance and 38 cm under SSD guidance. For BE, the mean buffer spaces were 43 cm under auditory guidance and 32 cm under SSD guidance. No significant differences were found for buffer space between blind non-echolocators and the sighted group under auditory guidance [*F*(1, 16) = 0.013, ns], or SSD guidance [*F*(1, 16) = 3.88, ns]. There was no effect of repetition. Buffer space for the sighted group under visual guidance was significantly smaller than for the blind non-echolocators under auditory guidance [*F*(1, 16) = 26.54, *p* = 0.001] or SSD guidance [*F*(1, 16) = 22.11, *p* = 0.001]. Modified *t*-tests showed that, on average under auditory guidance, buffer space was not significantly different for BE and the sighted participants (*t* = 0.16, ns) or the blind non-echolocators (*t* = 0.24, ns).

### Echolocation detection range

**Fig 6:**
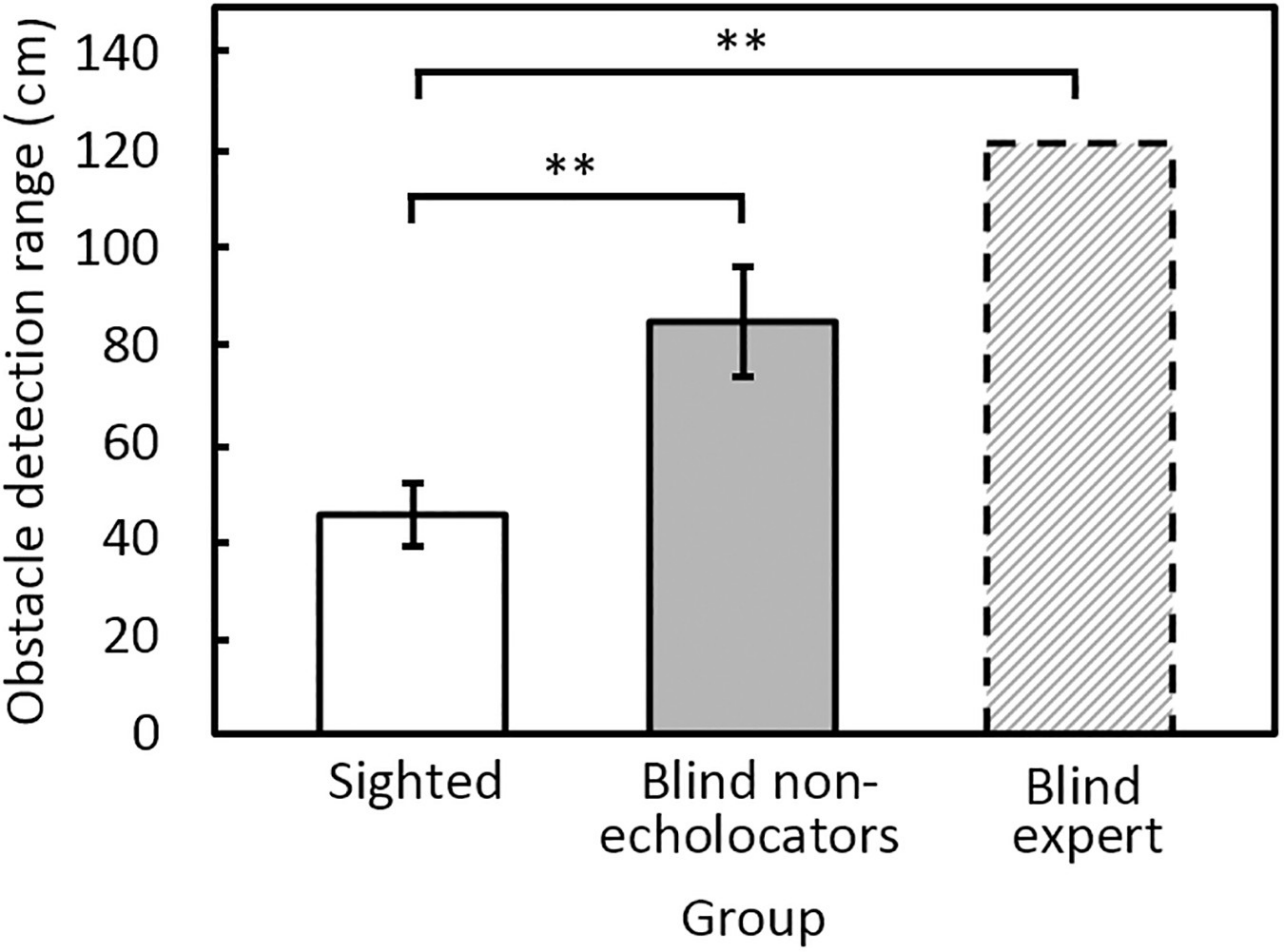
Mean echolocation obstacle detection range when navigating using audition.

Fig 6 shows that the echolocation obstacle detection range was greatest for BE and smallest for the sighted group, with performance for the blind non-echolocators falling between. Mean obstacle detection ranges were 45 cm for the sighted group; 85 cm for the blind non-echolocators, and 122 cm for the BE echolocator. The detection range was significantly greater for the blind non-echolocators than for the sighted group [*F*(1, 16) = 14.4, *p* = 0.002]. There was a significant interaction between repetition and group [*F*(2, 32) = 8.93, *p* = 0.001]. However, post-hoc tests indicated that there were no significant differences across repetitions for either group. Modified *t*-tests showed that, on average, BE had a greater echolocation range than the sighted participants (*t* = 4.72, *p* = 0.001) but not the blind non-echolocators (*t* = 1.26, ns).

### Side of avoidance of the obstacle

**Fig 7:**
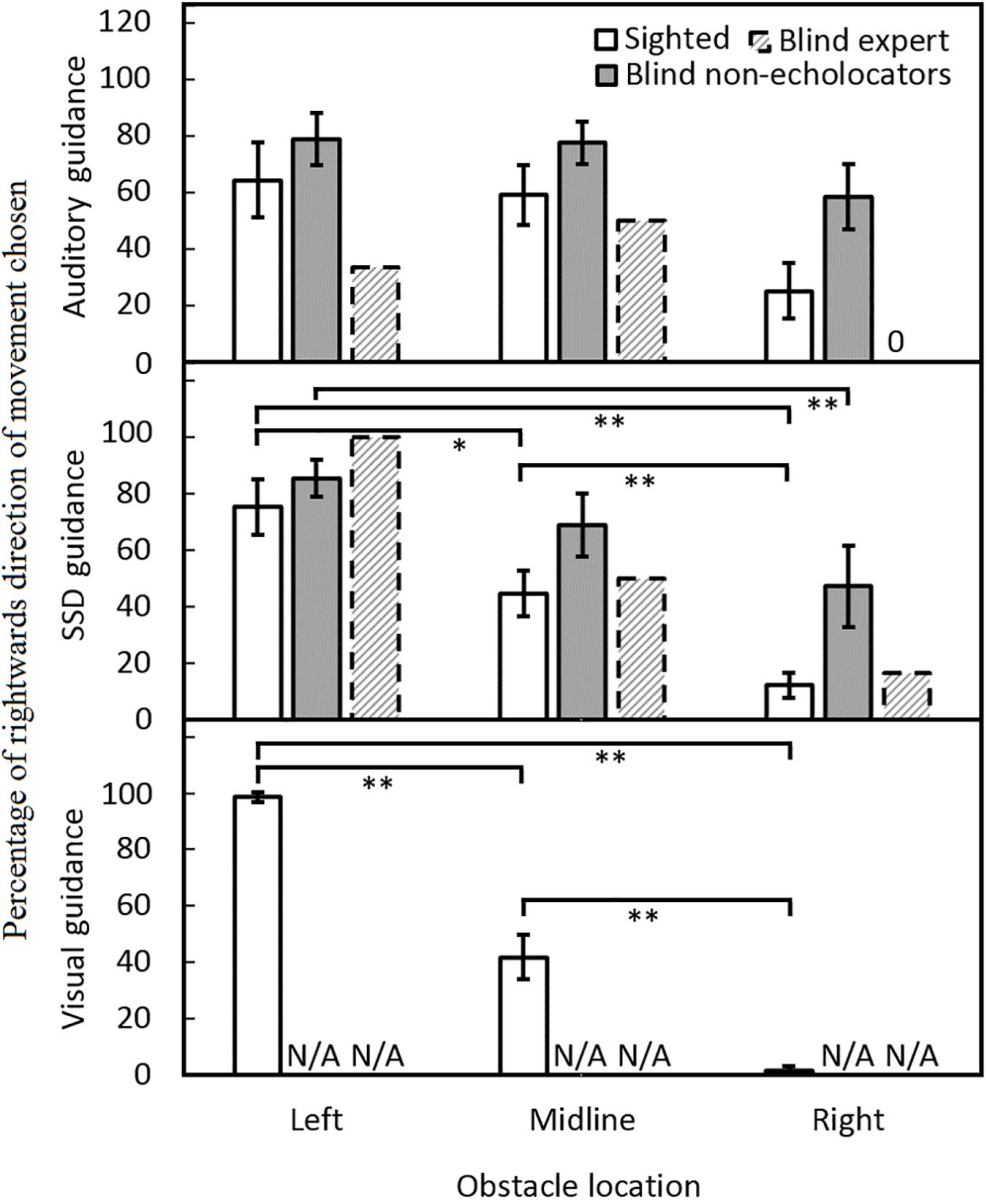
Percentage of times the participant chose to move in a rightwards direction to pass the obstacle under auditory guidance (upper panel), SSD guidance (middle panel), and visual guidance (lower panel), for sighted participants (open bars), blind non-echolocators (grey bars), and BE (dashed bars) for each obstacle location. Under auditory guidance, the BE echolocator made 0% passes on the right side of the obstacle when the obstacle was located on the right.

Fig 7 shows the percentage of times the participant chose to move in a rightwards direction to pass the obstacle. When navigating using vision, sighted participants nearly always moved past the obstacle on the side that afforded the most space, i.e. they moved in a leftwards direction when the obstacle was positioned to the right and vice versa (Fig 7, bottom panel). There was a trend in the same direction under SSD guidance for all groups, but the effect was markedly reduced under auditory guidance (Fig 7 middle and upper panels, respectively). The blind non-echolocators did not always move past the obstacle on the side affording most space, either under auditory or SSD guidance, whereas the sighted group generally did move past the obstacle on the side affording most space under SSD guidance. An analysis of side of avoidance for the sighted group showed a main effect of obstacle location only [*F*(2, 18) = 35.59, *p* = 0.001] and a significant interaction between obstacle location and guidance condition [*F*(4,36) = 4.93, *p* = 0.003]. Sighted participants moved rightwards to pass the obstacle significantly more often in conditions when the obstacle was positioned on the left or on the midline than when it was positioned on the right, and when the obstacle was positioned on the midline compared to being positioned the right, under visual guidance (*p* < 0.001), and SSD guidance (*p* < 0.016), but not auditory guidance. For blind non-echolocators, an analysis of side of avoidance showed a main effect of obstacle location only [*F*(2, 14) = 5.12, *p* = 0.02]. Blind non-echolocators moved rightwards to pass the obstacle significantly more when the obstacle was positioned on the left, than when it was positioned on the right under SSD guidance only (*p* = 0.01). Modified *t*-tests did not show any significant differences in side of avoidance between BE and the sighted group or blind non-echolocators (all *p* > 0.05).

## Discussion

The main findings were: 1) Under auditory guidance, the blind non-echolocators were better able to use echolocation in an obstacle circumvention task than the normally sighted group, as indicated by fewer collisions, lower movement times, fewer velocity corrections, and greater range of obstacle detection. However, buffer space was similar for both the blind non-echolocators and the sighted groups, and blind non-echolocators did not use sound to pass the obstacle on the side affording most space. 2) Under SSD guidance, navigation performance was generally comparable between the blind non-echolocators and the sighted group, indicated by similar numbers of collisions, movement times, velocity corrections, and buffer space. The sighted group generally moved past the obstacle on the side affording most space under SSD guidance, which the blind non-echolocators did not always do. 3) For sighted participants, obstacle circumvention was generally better under SSD guidance than under auditory guidance, indicated by fewer collisions, faster movement times and fewer velocity corrections, although buffer space was similar. For the blind non-echolocators, obstacle circumvention performance was generally similar under SSD and auditory guidance conditions, with similar numbers of collisions, movement times, velocity corrections and buffer space.

Navigation performance using auditory guidance was generally better for blind than for sighted participants, which is consistent with the perceptual enhancement hypothesis. In line with this, the blind non-echolocators were also better than the sighted group at detecting the obstacle using echolocation given comparable training, in the initial obstacle detection task prior to the auditory testing phase, and detecting the obstacle in the “no-click” control experiment. Navigation performance for BE using sound was slightly but non-significantly better than that for the blind non-echolocators, but it was significantly better than for the sighted group. The similar performance of BE and the non-expert blind participants suggests that expertise was not the primary factor driving obstacle circumvention using sound echoes. However, we acknowledge that a larger sample of blind experts in future studies is needed to assess this conclusion more rigorously. Finally, as anticipated, navigation performance was much better under visual guidance than under auditory or SSD guidance.

The exact way in which the blind participants differed from the sighted participants in their ability to use auditory cues for navigation remains unclear. Blind participants may have been better able to use cues such as the time delay of the echo produced by the obstacle, as well as ‘echoic tau’ [57] or ‘echoic time-to-contact’ [8]. The derivation of echoic tau involves the use of the rate of change of acoustic cues, such as intensity differences between the self-generated click and the corresponding echo, the delay of the sound echo, the changing angle of incidence of the sound echo, and changes in spectrum. It has been shown that blindfolded sighted participants who were moving were more accurate than static participants when judging the position of a removable wall using echolocation [35], suggesting that movement provides benefits for blindfolded sighted people. Wallmeier and Wiegrebe [25] showed that self-motion benefitted echo-acoustic orientation by sighted echolocating participants. The benefits may be even greater for blind people, but this remains to be determined.

Representation approaches to sensory processing (also known as model-based control) [58, 59] are based on the assumption that sensory information is used by individuals to form an internal representation of the local environment. Consistent with this, Milne, et al. [29] presented evidence suggesting that self-generated sounds could be used to form perceptual representations of the shapes of objects. Serino, et al. [60] pointed out that sighted people experience their bodies as being positioned at a specific location in the world, with a single visuo-spatial perspective, obtained using perceptual information regarding the position and orientation of the body in relation to external space. The blind participants in the current study may have been more accurate than the sighted participants in their perception of the spatial extent and position of their own bodies, helping them to avoid collisions when navigating. The results from the current study are consistent with the idea that blind people can use sound to perceive their location relative to external space and to construct an internal representation of an obstacle in the environment. Consistent with this, several of the blind participants reported that the obstacle was perceived as being “out there”.

The finding that blind and sighted individuals used spatial information from auditory and tactile inputs to guide navigation around an obstacle is consistent with the idea that the representation of space is amodal [44, 61, 62], and the concept of crossmodal functional parity (that sensory modalities sharing a task-based mechanism display a form of equivalence in perceptual behavior [45]). Although navigation using auditory and tactile information was poorer than that using vision, our data show that the information needed to perform the obstacle circumvention task was available in the auditory and tactile modalities as well as in the visual modality. Furthermore, information in the auditory modality was used more effectively by the blind than by the sighted participants, consistent with earlier studies [2, 3] and with the idea that prolonged reliance on sound leads to a more precise sound-evoked brain representation of the spatial environment.

An alternative approach to sensory processing is information-based control [63–65]. According to this approach, on-going sensory information is used to guide locomotion around an obstacle, rendering path planning or internal representations unnecessary. Blind participants may have been better able than sighted participants to adapt their movements “online” [65], because of their superior ability to utilize information from echoes. Our results do not rule out this alternative approach.

Cross-modal plasticity can result in the recruitment of visual navigation areas following blindness. Kupers, et al. [41] tested blind and blindfolded normally sighted participants using the TDU tactile SSD mentioned in the introduction, in a virtual route recognition task. They showed that in congenitally blind participants the posterior parahippocampus and posterior parietal and ventromedial occipito-temporal cortices were activated. These brain areas are involved in spatial navigation using vision, suggesting that following blindness cross-modal plasticity results in the recruitment of visual navigation areas. Fiehler, et al. [28] investigated the neural activity underlying echolocation-based processing of path direction by presenting binaural recordings of sounds produced during walking to blind echolocation experts and blind and sighted novices. They found activation in the superior parietal lobule and inferior frontal cortex in each of the three groups. For sighted echolocation novices, additional activation was found in the inferior parietal lobule and in the middle and superior frontal areas. These results raise the possibility that blind people may more spontaneously allocate directional meaning to echoes during locomotion, whereas sighted echolocation novices may need to use high-level spatial processing at a more conscious level to allocate directional meaning to echoes. In the current study, application of more conscious, high-level spatial processing may have contributed to increased movement times, velocity corrections and number of collisions for the sighted group relative to the blind group when navigating under auditory guidance.

Our results are consistent with the idea that blind individuals can utilize echoes to generate personal space, in a similar way to vision [46, 47]. Greater buffer space under auditory than under visual guidance suggests that even though blind participants were better able than sighted participants to use echoes for navigation, both blind and sighted participants generated a larger ‘protective envelope’ when walking using auditory guidance. Participants circumventing the obstacle using full vision almost always passed the side of the obstacle that afforded the most space, supporting previous findings [46, 63]. This also occurred for sighted participants navigating under SSD guidance, but not auditory guidance. The blind non-echolocators displayed a bias towards moving towards the right around the obstacle, for both auditory and SSD guidance and for all obstacle positions. The reason for this is unclear. However, all but one of these participants were right-handed. It is possible that blind non-echolocators, if not specifically instructed to pass on the side affording most space, prefer to move around the obstacle on their non-dominant side, so that they can react with their dominant arm should a collision occur. Further testing with a left-handed group would be needed to test this idea. A number of factors might influence the direction of travel around the obstacle, including the foot with which blind non-echolocators preferred to lead and the direction in which they chose to lean/orientate their head when they first explored the obstacle.

Although not reaching performance levels comparable to those obtained with visual guidance, auditory performance by BE was generally high across kinematic measures, with almost no collisions, relatively fast movement times, and few velocity corrections. This participant often performed better than the other blind participants, although the differences failed to reach significance. The trend towards better performance by BE suggests that perceptual enhancement following blindness and the effects of training might combine rather than being mutually exclusive. It is likely that training enhances the ability to use perceptual information effectively, and that this effect adds to the effects of perceptual enhancement. However, the inclusion of a single blind expert echolocator, and differences between her and the blind non-echolocators, including age differences and being left-handed instead of right-handed, limit the generality of the findings. It would be informative to conduct further tests with a larger number of blind expert echolocators. Good echolocation may be beneficial when calibrating auditory space [9, 31, 66]. Vercillo, et al. [31] showed that the perception of auditory space was improved for blind expert echolocators relative to blind non-echolocators in a space bisection task, most likely through audio-motor feedback that aided in sensory calibration.

Performance varied markedly across the blind non-echolocators under auditory guidance, with some showing performance similar to that of BE (BE, B1-2 and B4 made zero or only 1 collision across all trials), even though they had received no echolocation training. Similar individual differences have been reported previously for blind participants who had not received formal training when performing an echolocation task [6], and when performing other auditory spatial tasks, such as monaural sound localization [67, 68]. The reasons for the individual differences are not well understood. Possibly, some blind people implicitly learn to use information from echoes even when not trained to do so. Differences in age may also have contributed to the variability in performance across the blind non-echolocators. Increasing age has previously been shown to be associated with a decline in performance when navigating [69]. Levy-Tzedek et al. [70] showed that older sighted participants took longer than younger sighted participants to navigate a virtual maze using auditory cues, and the former also made more pauses and collisions. Age may also have contributed to the differences between the blind non-echolocators and BE, who was younger. However, the blind non-echolocators who performed similarly to BE (B1-2 and B4) were among the older participants in this group. Also, there was no correlation between number of collisions and participant age (*r* = 0.01) for the blind non-echolocators. However, there was a weak correlation between number of collisions and age of onset of blindness (*r* = 0.45, *p* = 0.27).

The current findings show similar performance for the blind non-echolocators and sighted participants when navigating using an SSD around a large obstacle, suggesting that blindness does not necessarily result in perceptual enhancement for tactile machine-produced echolocation. This appears to be inconsistent with previous studies showing better performance for blind than for sighted participants when navigating using the TDU SSD [40, 41]. However, a study that tested navigation and obstacle avoidance using the more minimalistic EyeCane SSD, a device designed to have low complexity to make it simple and intuitive to use with minimal training in common with the Miniguide used in the current study, showed similar performance for blind and sighted participants [71]. The difference across studies might reflect the limited amount of training given in our experiment, differences in obstacle size, or differences in the complexity of the devices and tasks used. Kupers et al. [41] reported that blind participants were better at using the TDU for a virtual navigation task only at the end of extended training, and that performance on the first day of testing was similar for blind and sighted participants. Also, no difference in performance between groups was observed for route navigation and recognition tasks. Chebat et al. [40] showed that blind participants were better than sighted participants at using TDU information to detect and avoid small obstacles. However, the two groups were equally good at detecting large obstacles. Further testing using the Miniguide SSD would be informative in establishing whether differences in performance arise between blind and sighted participants following more extensive training and for obstacles of smaller sizes. This could lend support to the hypothesis that the effects of perceptual enhancement and training combine, as described above.

The present experiments were conducted in a quiet environment with a single, static and highly reflective obstacle, and the walking speed was not restricted, making the task relatively easy. Increasing the task difficulty may lead to increased differences in performance between trained and untrained blind participants. Further work is needed to investigate locomotion in more challenging everyday environments, such as noisy rooms with high reverberation, where multiple dynamic objects are present that have higher or lower acoustic reflectivity, or when participants are moving at different walking speeds. Gérin-Lajoie, et al. [47] reported that under visual guidance, the shape and size of personal space were maintained at different walking speeds. However, as the spatial detail provided by sound and SSD devices is lower than for visual information, locomotor accuracy and efficiency based on sound or tactile information alone might be impaired when walking quickly.

The current results show that early-onset blind participants were able to use auditory guidance for obstacle circumvention more effectively than blindfolded sighted participants. However, further work is needed to establish whether people with late-onset blindness are able to use auditory guidance as effectively. Duration of visual loss has previously been shown to affect human movement performance [72] and echolocation acuity [19]. A study on the reaching and grasping performance of partially sighted individuals showed that the performance was better if the duration of visual impairment was longer [72]. For echolocation, Teng, et al. [19] reported a correlation between echolocation acuity and age of onset of blindness. If better echolocation acuity is linked with improved locomotor precision, people with late-onset blindness may perform more poorly at obstacle circumvention using auditory guidance than people with early-onset blindness. This remains to be tested. A survey showed that blind people who used echolocation found it easier to move around in novel places than those who did not echolocate, although echolocation benefits may have depended upon use of a long cane [73]. The results of the current study show that audition and echoic SSDs provide useful information for guiding navigation around obstacles. An investigation of the effectiveness of audition or an SSD when using a cane or walking with a guide dog would provide further information regarding the real-life practicality of these forms of guidance for blind people.

Obstacle circumvention performance was generally similar under SSD and auditory guidance conditions for the blind non-echolocators. These findings have practical implications for blind people, as a common question in relation to SSDs is whether it is worth buying a device and investing time to train with it if one can train to an equivalent or better level using self-generated sounds. SSDs have drawbacks such as cost and limited battery life, and training procedures for some SSDs are not well established [38]. Our findings suggest that even without training, using natural echolocation to circumvent obstacles leads to performance similar to that using an SSD. BE tended to move faster and more smoothly with fewer velocity corrections under auditory guidance than when using the SSD, but BE had much more experience of echolocation using self-generated sounds than using the SSD. One advantage of some SSDs is the adjustability of the detection range. This was set to 1 m for the current experiment, but the detection range for the Miniguide can be set as high as 8 m, which is greater than the detection range of the blind non-echolocators (approximately 0.9 m), and BE (approximately 1.2 m). Further investigation of the effects of training for both echolocation and SSD guidance, using different SSD ranges, would help to elucidate the advantages and disadvantages of the two modes of visionless navigation. The complexity of the task and environment are also factors that need to be explored to compare navigation under SSD guidance and echolocation.

## Supporting information

S1 File Participant data for number of collisions, side of obstacle avoidance, buffer space, overall movement times, the number of velocity corrections, and obstacle detection range for each guidance condition (audition, SSD and vision), for each obstacle location (left, midline and right). (XLSX)
